# Assessing development and climate variability impacts on water resources in the Zambezi River basin. Simulating future scenarios of climate and development

**DOI:** 10.1016/j.ejrh.2020.100763

**Published:** 2020-12

**Authors:** D.A. Hughes, F. Farinosi

**Affiliations:** a Institute for Water Research (IWR), Rhodes University, Grahamstown, South Africa; b European Commission, Joint Research Centre (JRC), Ispra, VA, Italy

**Keywords:** Zambezi river basin, Hydrological models, Climate change, Water use change, Model uncertainties

## Abstract

**Study region:**

: The Zambezi River basin, a transboundary basin supplying vital resources to vast human and environmental systems and subject to radical changes linked to climate and infrastructural development.

**Study focus:**

: Application of a hydrological model (Pitman) established for 76 sub-basins covering the total basin area of about 1 350 000 km^2^ to assess the potential impacts of increasing water demand under global warming scenarios (1.5, 2, and 3 degree).

**New hydrological insights for the region:**

: The application of the calibrated model to the analysis of different combinations of climate change and water use showed that the relative impacts are quite different across the whole Zambezi River basin. The greatest impacts are found in the areas containing large open water bodies (natural and man-made), that are very sensitive to the multiple effects of increased aridity. The uncertainty in the future simulation results remains hugely dependent upon the source of the climate change data and the change signals given by them. The sample RCM data (6 models) used are representative of many more model outputs, while the spread of possible climate change signals remains quite large. However, the main uncertainties do not invalidate the overall message of possible water resources change that is summarized in a substantial decrease in water availability under all the combined scenarios.

## Highlights

• Analysis of increasing water demand under 3 different degrees of climate change for the Zambezi basin.
• Even under the ambitious climate change management targets, water availability in the basin is estimated to decline.
• Large impacts in the Lake Malawi/Nyasa sub-system and in other areas with open water bodies.

## 1 Introduction

The introduction to the first part of this study refers to the ultimate aim of hydrological models, which is to improve the decision-making capacity of water management authorities (Hughes et al., 2020). One of the key issues for future water management is related to the likely impact of climate change on the natural water resources availability, as well as on the economic activities that rely and utlilise these resources (Milly et al., 2008). The other paper also emphasised the uncertainties inherent in establishing any hydrological model, some of these uncertainties being related to the imperfections of the models themselves, others to the accuracy and representativeness of the climate data used to force the models, and yet others related to the availability and accuracy of the observed stream flow data that provide the calibration signals used to parameterize the model and validate the outputs. In most large basins throughout the world, the stream flow data often reflect non-stationary impacts of water abstractions or modifications to the flow regime (e.g. through storage and hydro-power releases). All of the uncertainties associated with the data used to force, calibrate and validate a model are exacerbated in data scarce regions such as Africa. However, the uncertainties associated with setting up a model to represent historical flow regimes can pale into insignificance compared to those associated with predicting what the future may look like (Giorgio et al., 2009; Hargreaves and Annan, 2014; Wang et al., 2015). The uncertainties related to the various emission scenarios, general circulation models and their configuration, the downscaling approaches used (Shamseddin and Chaibi, 2019), as well as future socio-economic development considerations all have to be added to the model setup uncertainties.

There are many different ways in which the future uncertainties can be represented in a model study, but the most common approach is to generate an ensemble of model outputs, based on an ensemble of model inputs (Dosio et al., 2019). This ensemble of inputs includes all the time series data representing future possible climate situations, the uncertainty ranges in any of the model parameters (Erfanian et al., 2016) that are considered likely to be affected, as well as uncertainty ranges in the water use inputs (storage volumes, abstractions, artificial releases, etc.). However, given the large number of possible model inputs, particularly related to climate model simulations, it is also necessary to establish an appropriate approach for defining the input ensemble (Lutz et al., 2016). It is also necessary to decide how the output ensemble information should be presented to those who might use the information for decision-making purposes. It is not always easy to convey uncertain prediction results to an audience that is required to make decisions (Borgomeo et al., 2018; Greve et al., 2018), however, it is also not valid to simply present a multi-model, or ensemble, mean, particularly when all of the input ensembles are considered to be equally likely to occur.

The companion of this study (Hughes et al., 2020) described the hydrological models used to establish a basis for water resources assessments across the whole of the Zambezi River basin, outlined the model calibration approach and presented the results compared to the available observed stream flow data. The stated purpose of establishing the models is to establish an approach that allows for future water resources availability, under different scenarios of both development and climate change, to be assessed. The first part highlighted a number of uncertainties in the available data, the models used and our understanding of the hydrological dynamics of the basin, but concluded that the model setups (particularly the Pitman model) have been established with sufficient confidence and are fit for the purpose of assessing future changes. This paper therefore takes the next step and applies one of the established models (Pitman) to evaluate the range of possible conditions that might occur into the future, given up-to-date estimates of what that future might look like from both the perspective of the climate, as well as expected water consumption.

## 2 Study area

**Fig. 1:**
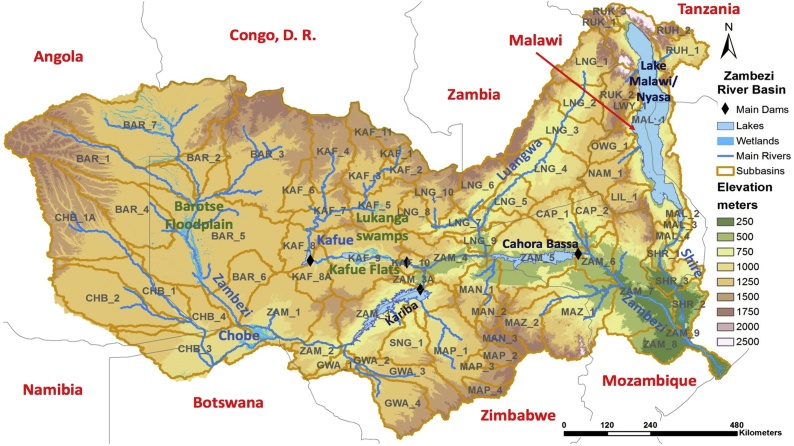
Map of the Zambezi River basin showing the main tributaries, sub-basin division and large wetlands and reservoirs.

The Zambezi catchment drains an area of 1 350 000 km^2^ crossing the borders of eight countries (Angola, Botswana, Malawi, Mozambique, Namibia, Tanzania, Zambia and Zimbabwe) in Central Southern Africa. Beside the main river, the system includes major tributaries such as the Luangwa, Kafue, Chobe, and Shire rivers; natural lakes such as Lake Malawi/Nyassa; artificial reservoirs such as Itezhi-Tezhi, Kafue, Kariba, and Cahora Bassa; and several wetlands (Luangwa and Barotse floodplains, Lukanga swamps, Kafue flats) (Fig. 1). The rainy season is concentrated in the summer, 4–6 months between October and March, precipitation ranges from over 1 200 mm y^−1^ in some of the headwater areas of the Shire and Kafue sub-basins, to less than 700 mm y^−1^ in the more arid parts of Zimbabwe (Hughes et al., 2020).

The main economic activities are represented by mining, agriculture, fisheries, tourism, and manufacturing. The basin hosts a population of about 40 million (ZAMCOM et al., 2015), with per capita water consumption estimated to be around 100 m^3^ yr^−1^, on average. The most water intense sector is agriculture, while small-scale rainfed agriculture represents the main source of income in the majority of the basin. The World Bank (2010) estimated agricultural land in the basin to be about 5.2 million hectares, with only about 0.25 million irrigated. Irrigation infrastructural development was identified as a priority and an additional 0.51 million hectares were already identified.

The hydropower potential of the basin was estimated to be about 20 GW, of which only about a quarter is currently exploited, mainly in the Cahora Bassa, Kariba, Itezhi-Tezhi, and Kafue Gorge dams in Zambia and Mozambique. About 40 additional possible installations (summing up to about 13 GW of installed capacity) have been already identified in the basin. Some of these projects are currently under construction within the Southern African Power Pool (SAPP) development programme. Several water transfer projects have also been planned for the basin (World Bank, 2010).

## 3 Methods and data

The companion paper to this contribution provides a brief description of the hydrological models that have been calibrated for the Zambezi River basin (Pitman and WEAP). For the future projections, only the Pitman model is used to simulate a number of future scenarios (Table 1). The first group of scenarios (S1, S1A and S1B in Table 1) are based on the historical climate information and use revised estimates of present day water use as well as uncertain estimates of future water use. The other three sets of scenarios are based on future estimates of climate conditions using 6 downscaled RCM outputs to represent projected conditions for the Zambezi basin under 1.5°, 2.0° and 3.0° average global temperature increases (S2, S3 and S4, respectively in Table 1).

**Table 1:** Definition of scenarios.

Scenario	Definition
S1	Single run with CRU rainfall and historical reservoir releases (Kariba and Cahora Bassa) replaced with fixed operating rules.
S1A	Same as S1, but with improved estimates of present day water use.
S1B	Same as S1, but with uncertainty estimates of future water use.
S2.1A	Ensemble rainfall inputs (1.5° temperature scenario) & present day water use.
S2.2A	Same as S2.1A, but with changed evapotranspiration for 1.5° temperature scenario.
S2.2B	Same as S2.2A, but with estimates of future water use.
S3.2B	All climate change effects for 2.0° temperature scenario & future water use.
S4.2B	All climate change effects for 3.0° temperature scenario & future water use.

### 3.1 Climate data preparation from RCM’s

Climate scenarios for the analysis were derived from the COordinated Regional-climate Downscaling EXperiment (CORDEX) project (Giorgi et al., 2009) downscaled Regional Climate Models (RCMs) outputs. The overall CORDEX project goal was to dynamically downscale the results of Global Circulation Models (GCMs) participating in the Fifth Coupled Model Intercomparison Project (CMIP5, Taylor et al., 2012) to produce high resolution historical and future projected data able to more accurately reproduce the regional and continental climate dynamics. The reason to prefer a dynamic downscaling methodology, instead of a statistical one, is related to the ability of a RCM to better reproduce the peculiarities of a regional setting respect to a statistical methodology relying on ground observations, very scarce for the area under consideration. In addition, dynamical downscaling methodologies have been proved to be able to more accurately maintain the range of variability of the original GCM respect to the statistical ones (Ito et al., 2020). The CORDEX-Africa project made use of 5 different RCMs downscaling the results of 10 GCMs, for a total of 23 combinations. Out of the 23 datasets available, in this analysis, we selected a set of 6 different modeling outputs (Table 2) derived from the combination of 3 RCMs (CLMcom−CCLM4.8–17, GERICS-REMO, and SMHI-RCA4) and 5 GCMs (CNRM-CM5, HadGEM2-ES, MPI-ESM-LR, MIROC5, and IPSL-CM5A-MR). The model outputs were selected to cover the largest variability of the climate conditions and to include the largest range of uncertainty spanned by the RCMs for the region under consideration. When selecting the models, our attention focused particularly on the comparison of the historical precipitation seasonal means and intensities with respect to the observed data, as made available by two recent studies (Dosio et al., 2019, 2020). Original data for the 6 selected combinations, were made available at a spatial resolution of 0.44 degree (∼48 km at the equator) at daily time steps from the Earth System Grid Federation (ESGF). Climate projections from different models reach different global average temperature levels in different times due to the specific GCM’s characteristics. In this analysis, we decided to focus on climate scenarios compatible with the Paris Agreement (UNFCCC, 2015 and 2016). In the 2015 Conference, the State Parties committed to keep global temperature increase below 2 °C, with more emphasis on an even more ambitious 1.5 °C target. Therefore, we selected these two thresholds and an additional scenario (3 °C) representing the possible failure of the implementation of the Agreement. In order to represent the 1.5, 2, and 3 °C scenarios, we selected the 30-years data centered in the year in which the specific model (Table 2), in its RCP 8.5 scenario, reaches the warming threshold selected as reported in Dosio and Fischer (2018) and Farinosi et al. (2020). It has to be noted that the 1.5, 2, and 3 °C global average warming scenarios, might result in different levels of local temperature changes in different parts of the world.

**Table 2:** Climate models used in the assessment and the 30 year periods used to represent the three global warming scenarios.

	Central year corresponding to the warming level of		
Model	1.5 degree	2.0 degree	3.0 degree
**CCLM_CNRM_CM5**	2029	2044	2067
30-years range	(2015−2044)	(2030−2059)	(2053−2082)
**CCLM_HadGem2-ES**	2018	2030	2051
30-years range	(2004−2033)	(2016−2045)	(2037−2066)
**CCLM_MPI-ESM-LR**	2028	2044	2067
30-years range	(2014−2043)	(2030−2059)	(2053−2082)
**RCA4_MIROC5**	2027	2043	2067
30-years range	(2013−2042)	(2029−2058)	(2053−2082)
**REMO_IPSL-CM5A-LR**	2022	2035	2054
30-years range	(2008−2037)	(2021−2050)	(2040−2069)
**REMO_MIROC5**	2027	2043	2067
30-years range	(2013−2042)	(2029−2058)	(2053−2082)

### 3.2 Climate data pre-processing for rainfall inputs to the model

Hughes et al. (2014) refer to a previous approach to incorporating climate change rainfall uncertainties into a hydrological model, using rainfall data for nine statistically downscaled GCMs, for baseline (1961–2000), near-future (2046–2065) and far-future (2081–2100) periods. Given that the nine baseline rainfall simulations were very different from the available historical data, a bias correction approach was applied to generate corrected future rainfall time series used to re-run (9 times) a model previously calibrated using historical rainfall data. Hughes et al. (2014) referred to a number of possible problems with this approach and proposed an approach that uses a pre-processing routine to quantify the uncertainties in the changes between the baseline and future rainfall patterns across all of the climate models and generate stochastic rainfall ensembles used to force an uncertainty version of the hydrological model. The pre-processing program used in this study calculates the annual means and standard deviations for the baseline and three future (30-years, representative of 1.5°, 2.0° and 3.0° temperature increases) periods for each climate model. The delta change (as fractions) in annual means (ΔM_i_) and standard deviations (ΔSD_i_) are then calculated for each RCM (i) and their ranges determined across all available RCMs (ΔMmin to ΔMmax and ΔSDmin to ΔSDmax). The mean calendar month fractions (of the annual total) and their delta changes (between the baseline and three futures) are also calculated for each period and climate model combination to generate similar minimum and maximum ranges across the RCMs. Uniform distribution random samples from these ranges are used to generate delta change values which are applied to the historical rainfall time series used for the original calibration of the model (CRU data for 1901–2017) to generate 250 stochastic rainfall ensembles. The full set of algorithms for the approach are given in the appendix. The random numbers used for the delta change samples of annual mean, annual standard deviation and monthly fractions are independent of each other, but each sample value is applied to the whole time series of a single ensemble. The final part of the algorithm re-calculates the monthly rainfalls in any one year to make sure that the annual value is correct after the monthly fraction delta changes are applied (which may not necessarily add up to 1). The same random number sample value is applied to all of the sub-basins within a single ensemble to ensure that the whole basin is represented by a single possible climate change signal. However, it is important to note that regional differences in delta change signals (e.g. some areas expected to become wetter or drier than other areas under the same climate change scenario) will be preserved because the sampling is based on the delta change ranges for each individual sub-basin.

The key issue is that changes in the annual means and standard deviations are considered to be independent across the RCMs, as are the seasonal distributions of rainfall. Because the delta changes are applied to the original historical rainfall data (CRU in this study), the method also assumes that the historical sequences of wet and dry periods will be more-or-less preserved into the future and this was previously identified as being reasonably valid for South Africa (Hughes et al., 2014). A preliminary analysis of the data for the 6 climate models did not reveal any systematic relationships between the changes in annual means and seasonal distributions, supporting the use of independent sampling from the different delta changes. It is also important to note that any inter-annual variations in seasonal distribution (related inter alia to variations in the movement of the ITCZ) present in the historical rainfall data will be largely preserved using the method applied.

### 3.3 Climate data pre-processing for evapotranspiration inputs to the model

The evapotranspiration data, the main climate input to the PITMAN model together with precipitation, were estimated using the LISVAP pre-processor (Burek et al., 2013). LISVAP is the module of the distributed hydrological model LISFLOOD used to estimate potential Evapotranspiration (ET_0_) and the potential Soil (ES_0_) and Open-Water (EW_0_) Evaporation. The potential ET values are computed applying the Penman-Monteith (P-M) equation and theoretically estimated considering a hypothetical reference short crop with approximate height of 0.12 m with unlimited availability of water (as in Allen et al., 1998). In order to estimate the ET_0_, both for historical and projected climate data, the P-M method uses daily values of several variables: minimum and maximum near-surface temperature, near-surface specific humidity, surface air pressure, surface downwelling and upwelling shortwave and longwave radiation, and near-surface wind speed. As showed in recent studies (Naumann et al., 2018 and Dewes et al., 2017), the P-M method provides the most accurate estimates of potential evapotranspiration for the climate projections. Similarly to the process adopted for the precipitation estimates, projected ET_0_ from the 6 RCM’s, for each of the 30-year ranges associated with the three warming levels selected, were compared to the historical portion of the RCM itself to estimate the delta changes. Delta change is calculated using the mean and standard deviation for each RCM analyzing both annual totals and monthly distributions. The delta changes were then applied to the historical ET data used for the original model calibration (Hughes et al., 2020) consistent with the hydrological model requirements. The final input to the hydrological model is a range of uncertainty (minimum and maximum values) in the annual potential evapotranspiration value for each warming level, while the seasonal distributions used for the scenarios remained the same as the historical data (the analysis of the RCM’s did not suggest significant changes in the seasonal distributions). It is important to note that when the model runs through the ensembles, the uncertainty range is randomly sampled (see later section on ‘Running the model’). However, it would not be correct to allow the random sampling to occur independently across all of the sub-basins, as this would mean that any single ensemble would have a wide mixture of possible ET_0_ change signals. Therefore, all sub-basin ET_0_ samples are drawn from the same part of the delta change range within any single ensemble.

### 3.4 Estimating present day and future water use

The initial calibration of the model (Hughes et al., 2020) did not take into account all of the present day water uses, largely because the calibrations were based on historical observed stream flow data that may not reflect current levels of water use. There is very little information available to directly quantify water uses, and this is one of the typical data scarcity problems that is prevalent throughout southern Africa. When estimating water uses we have to identify how much water is used, what the water is used for (in order to estimate the seasonal distributions of use), as well as the source of the water (large dams, small distributed dams, direct river abstractions or groundwater). The dominant consumptive water use (i.e. excluding hydro-power) is expected to be for irrigation from distributed small to medium sized reservoirs (< about 5 * 10^6^ m^3^ maximum storage), as well as a few larger dams (mostly within the Zimbabwean sub-basins). The areas under irrigation are quantified by intersecting a raster coverage (IFPRI, 2019: ∼100 km^2^ grid size) of irrigated areas with the polygon coverage of sub-basins to extract the total number of hectares per sub-basin. The same data source can be used to estimate the total area of rain-fed agriculture. The latter is expected to influence the downstream flow regimes through changes to runoff generation processes, but these impacts are assumed to have existed for many years and would have been accounted for in the hydrological model calibration (albeit implicitly rather than explicitly). The estimated irrigation extents were then partially ‘ground-truthed’ by estimating the areas of obvious irrigation (mostly by identifying centre pivot sites) from Google Earth, as well as using some local knowledge in parts of the Luangwa River sub-basins (Lawrence, personal communication, 2020)^1^ . Lawrence (2020) also provided some insight into the crop types that might be expected, from which we could approximately estimate the seasonal distribution of water demands. The methods used to estimate water use ignore any use for mining purposes which are known to exist in parts of Zambia (Copper Belt) and Zimbabwe. Unfortunately, we have very little information on these water uses, but they are not expected to very large, relative to the total streamflow in the relevant sub-basins.

The maximum surface area of reservoirs is based on a GIS coverage of water bodies (Pekel et al., 2016; Gonzalez Sanchez et al., 2020). This coverage includes wetland areas, as well as artificial storage and several processing steps were required to eliminate the former, before the coverage could be intersected with the sub-basin coverage to extract the total surface area of reservoirs and then estimate the total storage volume using assumed area-volume power relationships (Hughes and Mantel, 2010). The parameters used in the area-volume relationships are extremely uncertain, in the absence of reservoir bathymetric surveys, and this uncertainty can be very large for reservoirs with large surface areas. Fortunately, there are independent sources of volume data for at least some of larger reservoirs, notably the register of dams available from the FAO Aquastat database (http://www.fao.org/aquastat/en/databases/dams/, accessed during March 2020) such that these can be eliminated from the GIS analysis and their volumes added back afterwards. The results for the remaining dams will still be uncertain, but there are no other known sources of information available. To be able to use the total reservoir volumes in the hydrological model it is also necessary to quantify the proportion of the total sub-basin area that contributes to these dams. While in theory this could be done automatically (using a DEM and automatic delineation of catchment areas for each dam), this is not a practical proposition as there are many thousands of small dams. The proportions were therefore estimated subjectively by simply viewing the distribution of the dams together with the sub-basin areal extents and the available river channel coverage. Inevitably, the contributing proportion of the sub-basin becomes a highly uncertain parameter in the hydrological model (see next section for more details).

### 3.5 Running the model for climate and/or water use scenarios

There are several versions of the Pitman model available within the SPATSIM framework. The simplest is the single run version that was mainly used for the model calibration runs (Hughes et al., 2020). The single run version uses fixed parameter values (including annual potential evapotranspiration depth) and a single input of rainfall time series data. The other versions allow for uncertainty in the parameter inputs using minimum and maximum values to define uniform distributions, which are sampled during the ensemble model runs. For scenario 1B (Table 1), only the water use parameters are considered uncertain and the model generates 10 000 ensembles using independent random sampling of the range of future water use parameters. For scenario 2.1A, only 250 ensembles are generated and these are based on random sampling from the ensemble of stochastic rainfall inputs (explained above) with no additional parameter uncertainty. All of the *.2B scenarios (i.e. changes to rainfall, evapotranspiration demand and water use) are based on a combination of 250 rainfall input samples plus 200 samples from the parameters that are considered uncertain (annual potential evapotranspiration and future water demands), therefore generating 50 000 ensembles. A post-processing utility is used to extract the minimum, maximum and 95, 50 and 5% exceeded values for each month of the time series across all of the ensemble sets. The main purpose of this utility is to generate data that can be used to plot the ensemble bounds for any given scenario. The utility is normally used to extract the simulated cumulative flow at the outlet of any sub-basin, however, it can also be used to examine the uncertainty bounds of sub-basin incremental flow or the storage volume of a reservoir or wetland (for those sub-basins where they exist).

## 4 Water use scenarios (present day and future)

During the calibration phase of the project, some poorly defined existing water uses were largely ignored as they were not considered likely to affect the overall calibration, or were expected to have occurred more recently than most of the historical observed stream flow data that formed the basis of the calibration. However, it is important to include these in the present day scenario (S1A in Table 1), even if they remain poorly quantified. Table 3 summarises the estimates of irrigation water use based on the GIS analysis of assumed irrigation areas (IFPRI, 2019) and a fixed demand of 750 mm y^−1^. This value was based on some local knowledge of irrigation practices in parts of Zambia (Lawrence, 2020), while it might be expected that some of the drier parts of the basin (e.g. within Zimbabwe) will have higher demands.

**Table 3:** Irrigated and rain-fed (dryland) agricultural areas (those sub-basins not included have lower areas than the minimum category value; 26 and 8 for irrigation and rain-fed agriculture, respectively).

Irrigated agricultural area (ha) categories	Water use (m^3^ * 10^6^ y^−1^) based on 750 mm y^−1^ irrigation	Sub-basins included in category (in order of largest irrigation area)
> 10 000	> 75	MAP1, LNG10, ***MAL1***, KAF9, ***SHR3***, MAN2
5 000–9 999	37.5–74.3	MAZ2, MAN1, ZAM4, ***OWG1***, MAN3, ***ZAM8***
2 000–4 999	15.0–37.5	MAP2, LNG8, ***KAF3***, MAP4, MAZ1, ZAM3, KAF2, **BAR7**, ***KAF5***, **LNG7**, ***ZAM5***, **LNG3**
1 000–1 999	7.5–15.0	MAP3, GWA3, ***ZAM7***, ***LNG5***, ***ZAM1***, LNG4, ***KAF10***, ***NAM1***, ***KAF7***
500 to 999	3.75 to 7.5	***BAR6***, ***ZAM6***, **LNG2**, ***RUK3***, GWA4, ***LNG6***, ***LNG1***, ZAM2, ***KAF8***
100 to 499	0.75 to 3.75	***SHR1***, ***RUK1***, ***RUH1***, ***ZAM9***, ***ZAM3A***, **CHB3**, ***RUH2***, ***SHR2***
Rain-fed agricultural area (ha) categories		
>500 000		MAL1, NAM1, ZAM7, MAZ1, LIL1
200 000–499 999		RUK2, ZAM5, MAL4, MAP1, KAF9, OWG1, SHR3, LNG4, ZAM3, SHR2, SNG1, MAZ2
100 000–199 999		ZAM6, MAL3, LNG3, MAN1, RUH1, CAP1, ZAM8, RUK1, BAR5, MAL2, KAF5, LWY1, GWA3, SHR1
50 000–99 999		LNG8, LNG5, ZAM4, RUK3, LNG1, MAN3, ZAM2, MAN2, ZAM1, KAF2, BAR3, BAR7, MAP2
10 000–49 999		BAR4, LNG10, CHB1A, LNG7, KAF8A, GWA4, KAF11, BAR1, KAF3, ZAM9, KAF4, RUH2, ZAM3A, CHB3, KAF1, MAP3, BAR6, KAF10, BAR2, LNG6, LNG9, GWA1

*Notes*: Irrigation areas for sub-basins in ***bold and italic*** text are assumed to be supplied directly from rivers or lakes, while those in **bold and underlined** text could not be validated from Google Earth.

Table 4 lists the irrigation water demands for those sub-basins where there is also evidence of supply from either distributed small reservoirs or from large reservoirs, and includes the estimates of the reservoir full supply volumes. The last two columns in Table 4 provide an estimate of the sub-basin contributing area (%), as well as the mean annual inflow volume (m^3^ * 10^6^ y^−1^) based on the calibration simulations. There are some cases where there is a reasonable match between the estimated inflow volumes, the available storage and the demand (e.g. MAP1, MAN1, and MAP3), while there are other cases where there appears to be far more water available than the demand (e.g. LNG10, LNG8 and MAM2), as well as others where the demand appears to exceed the estimated supply (KAF9). Table 5 provides a summary of the water uses that were included in the calibration phase, as well as estimates for both the present day (S1A) and future (S1B to S4B) scenarios. This Table includes additional sub-basins where there are water demands assumed to be supplied directly from the river, as well as water demands for urban and mining supplies. The following sub-sections provide more information about the water uses and the information used to generate the estimates. It has to be acknowledged that the majority of the estimates in all the water demand tables (Table 3, Table 4, Table 5) are based on limited information, uncertain analysis methods, or subjective assessments of future demands. The full details of how the future demands are likely to be met are not included in Table 5 (lack of space), but the general assumption is that where reservoir storage is currently used, this will increase in the future.

**Table 4:** Estimated water demands, distributed reservoir storage and inflow estimates for those sub-basins mostly affected by irrigation demands fed by storage (all water volumes are in m^3^ * 10^6^). The large reservoirs that are not used for irrigation purposes are not included in the table.

Sub-basin	Water demand	Reservoir storage		% sub-basin area contributing	Annual inflow volume
		Small distributed	Large		
MAP1	110.4	194.3		25	180.9
LNG10	106.7	70.8		10	294.4
KAF9	98.7	84.0		5	34.1
MAN2	81.2	66.6	360.0	80	269.8
MAZ2	72.8	207.66		60	860.8
MAN1	65.6	91.0		30	120.7
ZAM4	62.1	100.8		10	49.5
MAN3	53.9	213.9		90	316.4
MAP2	34.1	35.2		30	92.6
LNG8	33.2	38.5		20	309.4
MAP4	31.5	125.3	154.0	90	200.3
MAZ1	31.2	83.2		15	261.4
ZAM3	28.5	11.0		5	74.1
KAF2	21.9	22.9		10	109.3
MAP3	13.3	13.9		15	18.1
GWA3	13.1	53.8		25	117.3
LNG4	9.9	9.3		5	94.7
GWA4	4.8	72.2		30	55.3
ZAM2	4.3	32.4		20	37.0

*Notes*: The large dams with ***bold and italic*** text are used for hydro-power, while those with **bold and underlined** are assumed to be used mostly for domestic or mining water supply.

**Table 5:** Present day and future water use estimates.

Sub-basin	Water use m^3^ * 10^6^ y^−1^			Purposes
	Calibration	Present day	Future	
LNG1 & 3	0	20.6	40.0	Direct irrigation.
LNG4, 5 & 6	0	25.7	50.0	Direct irrigation and from distributed small dams.
LNG8	0	33.2	60.0	
LNG10	0	106.7	150.0	
KAF11, 1 to 3	62.0	100.0	200.0	Direct irrigation, mining & domestic.
KAF5	0	18.2	36.0	Direct irrigation.
KAF6 to 8	2	11.8	25.0	Direct irrigation.
KAF9 & 10	0	146.0	260.0	Irrigation and urban water supply.
BAR6	0	7.0	10.0	Direct irrigation.
ZAM1	0	15.4	526.0	Direct irrigation & from distributed small dams. Also direct abstractions for urban supplies & a planned transfer to Botswana in ZAM1.
ZAM2	0	4.3	9.0	
ZAM3	0	28.5	60.0	
GWA3	0	20.0	40.0	Irrigation, mining & domestic use from small dams & GW.
GWA4	0	10.0	20.0	
MAP1	0	110.4	150.0	Irrigation & mining from distributed small dams & GW. There is a dam at the outlet of MAP3 supplying the Kadoma district.
MAP2	6.8	34.1	45.0	
MAP3	6.8	17.9	35.0	
MAP4	15.0	31.5	60.0	
MAN1	0	65.6	100.0	Irrigation & urban use for Harare in MAN3.
MAN2	0	81.2	120.0	
MAN3	114.0	83.9	250.0	
MAZ1	0	31.2	50.0	Irrigation & possibly some domestic use from distributed dams.
MAZ2	0	72.8	120.0	
MAL1	0	181.0	360.0	Direct from Lake Malawi/Nyasa.
LIL1	0	20.0	45.6	From a large reservoir.
SHR1	0	16.5	33.0	Direct from the river for urban supply.
SHR3	0	90.0	180.0	Direct from the river.
ZAM4	0	62.1	120.0	Irrigation from distributed small dams.
ZAM6	0	12.0	25.0	Irrigation & domestic, direct from river.
ZAM5, 7 & 8	0	74.0	150.0	Direct from the river.

### 4.1 Luangwa River

There is very little clear evidence of extensive cultivation or irrigation in the main eastern headwater areas (LNG1 to LNG5) nor in the downstream sub-basins (LNG7 and LNG9). The present day demand (Table 4) for areas LNG4, 5 and 6 are based on the IFPRI (2019) data, and is expected to double in the future. Both LNG8 and 10 have clearly identifiable irrigation areas, as well as extensive dryland cultivation. There is one large irrigation scheme upstream of Mita Hills reservoir (LNG10) that covers an area of greater than 1 500 km^2^, while estimates based on local knowledge (Lawrence, Pers. Comm, 2020) broadly agree with the IFPRI (2019) data that the area irrigated at any one time is less than 160 km^2^. It has been assumed that the area has almost reached full development (partly due to competition with future hydropower requirements) and will only expand to about 200 km^2^. Within LNG8 there is approximately 40 km^2^ of obvious irrigation (centre pivots) which is supported by a number of distributed small dams. The demand is assumed to double into the future. There are large reservoirs in both LNG8 (Mulungushi dam with storage of ∼490 * 10^6^ m^3^ and generating capacity of 32 MW) and LNG10 (Mita Hills dam with storage of ∼680 * 10^6^ m^3^ and generating capacity of 24 MW), while a future new storage reservoir is planned within LNG10 with additional storage of ∼450 * 10^6^ m^3^.

### 4.2 Kafue River

There are clear signs of irrigation and water use for mining (assumed to be direct from the river) in the headwater sub-basins (KAF11, 1, 2 and 3) above the Lukangwa swamps, and these are expected to increase into the future. There is less evidence of cultivation in KAF4 and future water use in this sub-basin is not expected to be significant. There are large areas of cultivation in KAF5 around the borders of the Lukangwa swamps and along the main river, with some indication of irrigation. Similarly, KAF6 and KAF7 have relatively small areas of irrigation. There is some cultivation around Itezhi Tezhi reservoir (within KAF8) as well as in the southern parts of the sub-areas representing the Kafue flats (KAF9), but this appears to be mostly dryland farming. There are two extensive areas of irrigation, close to the main river and near the outlet of KAF9 (just upstream of Kafue Gorge dam and close to Lusaka). The total area of irrigation for KAF9 is estimated by IFPRI (2019) data to be about 132 km^2^ and is assumed to be supplied direct from the Kafue River as well as from some reservoirs (Table 3). It has been assumed that the area will increase by about 50 %, a relatively low figure compared to assumptions in other sub-basins, largely due to the limited land space and competition with water for hydropower development. The Zambian capital, Lusaka lies on the catchment boundary between KAF9 and ZAM4 (the Kariba Dam sub-basin) and according to the web site of the Lusaka Water and Sewerage Company (http://www.lwsc.com.zm/; accessed in February 2020), some 36 * 10^6^ m^3^ y^−1^ is abstracted at the Iolanda water treatment plant just upstream of Kafue Gorge dam (in KAF10). This translates into about 50 L head^−1^ d^−1^ for the current population of Lusaka. Further information suggests that there is also some groundwater abstraction in the city itself. Clearly, both of these are expected to grow substantially in the future.

### 4.3 Upper Zambezi (Kariba and upstream) and Chobe River

While there is evidence of quite extensive cultivation in the eastern headwater tributaries above the Barotse floodplain (notably within BAR3 and BAR4), there is no evidence of irrigation. Further downstream there is cultivation along the main river and some irrigation (∼12 km^2^) near the town of Katima Mulilo (at the outlet of BAR6), but this is very small relative to even the dry season flow in the Zambezi at this point and is not expected to expand a great deal. While there is a planned hydro-power plant at Ngonye Falls (BAR5), this is a run-of-river scheme and is not expected to substantially influence the downstream flow regime. There is no evidence of much cultivation or irrigation in the sub-basins of the Chobe River. There is some irrigation in the tributary catchments of ZAM1, 2 and 3, as well as direct abstractions from the river in ZAM1 for several towns. There is also a proposed inter-basin transfer scheme to supply water from the Zambezi (within ZAM2) to Botswana at a rate of 495 * 10^6^ m^3^ y^−1^, as well as a further transfer scheme to supply water for a new dam (Shangani Dam) on the Gwayi River in Zimbabwe to be operated mainly for hydro-power. It has proved to be very difficult to obtain reliable values for these planned transfers and only the Botswana transfer has been included in the model at this stage.

### 4.4 Central basin tributaries

Some storage dams were allowed for in the calibration model for the Gwayi River sub-basins, but due to lack of information no water use was included. The expected water uses in GWA3 and GWA4 include those for agriculture, mining and domestic supplies and the IFPRI (2019) data are expected to under-estimate the total demand. Certainly, the available storage and inflow volume are far greater than the demand given in IFPRI (2019), even if the 750 mm y^−1^ of irrigation demand is increased for this drier region. Mkandla et al. (2005) provide some information about water supplies to Bulawayo (on the catchment boundary of GWA4) and the majority of the city supplies appear to come from surface water storage to the south (in the Limpopo River basin), but they do refer to some groundwater usage (3.6 * 10^6^ m^3^ y^-1^) from GWA4. GWA1, GWA2 and SNG1 are not expected to experience significant water uses, despite quite large areas of rain-fed agriculture.

The Senyati River sub-system (MAP1 to 4) has several large reservoirs (e.g. Claw Dam with 21 * 10^6^ m^3^ total storage and assumed to supply the Kadoma district with water) as well as a large number of distributed small reservoirs. The total storage appears to be over 500 * 10^6^ m^3^, but the water demand appears to be less than 200 * 10^6^ m^3^ y^−1^, possibly reflecting the semi-arid and variable nature of the flow regime. The Manyame River sub-system includes three large dams within MAN3 and one in MAN2, the most upstream one (Lake Chivero) supplying Harare with water. There are also a large number of much smaller dams in all the sub-basins with a combined storage of over 370 * 10^6^ m^3^ y^−1^. The total current irrigation demand is estimated at some 200 * 10^6^ m^3^ y^−1^ (Table 4, Table 5), while it is assumed that an additional 30 * 10^6^ m^3^ y^−1^ is abstracted for urban water use (a very uncertain value). Many of these demands are expected to increase in the future, particularly those from the large dams in MAN3 to supply the city of Harare. The headwaters of the Mazoe River (in MAZ2) lie just to the east of Harare and are intensively cultivated with many small to medium sized dams. The headwaters of MAZ1 also appear to be similar, but is perhaps less intensively cultivated. As with the other Zimbabwe sub-basins, future water demands are expected to increase, and while the level of increase is very uncertain, it is likely to be limited by the available resources. Given the current level of small dam development, it is not expected that the reservoir storage capacity will be increased substantially in the future.

### 4.5 Lake Malawi/Nyasa sub-system

There is extensive cultivation in many of the valleys of the headwater tributaries, as well on the shores of the lake. The IFPRI (2019) data suggest that there is about 240 km^2^ of irrigation, most of which occurs near the lake and can therefore be considered in the model as abstractions from the lake. There is also a quite large area of irrigation in SHR2 (120 km^2^). LIL1 contains Malawi’s capital city, Lilongwe (population of approximately 1.1 million), which is supplied by Kamuzu Dam (total storage of about 24 * 10^6^ m^3^ y^−1^ and a catchment area of 1 870 km^2^). The present day abstractions are based on 50 L head^-1^ d^-1^, and expected to increase in the future (Table 5). This information is based on data from Lilongwe Water Board (https://www.vei.nl/partners/lwb; accessed during March 2020). Blantyre (SHR1) is supplied with water direct from the Shire River. Current demand is estimated at about 16.5 * 10^6^ m^3^ y^−1^ and is expected to at least double into the future.

### 4.6 Main Zambezi River downstream of Kariba and other tributaries

There is some irrigation in the tributary areas of all the main Zambezi River sub-basins, with the highest being in ZAM4 and assumed to be supplied by the available reservoir storage. Further downstream most of the irrigation appears to be along the river. Some allowance has also been made for direct abstractions for the city of Tete (population ∼310 000) from ZAM6.

## 5 Results

The results for the different scenarios are mainly presented as flow duration curve comparisons at four key sites in the basin. These sites are the inflows to Itezhi Tezhi and Kariba dams (KAF7 and ZAM2), flows downstream of Cahora Bassa at Tete (ZAM6) and flows at the outlet of Lake Malawi/Nyasa (MAL1). Additional comparisons are made for some other sub-basins where these illustrate specific issues relating to water management under future projected changes. All of the future scenarios are compared with Scenario 1 that represents the calibrated model outputs but with fixed operating rules for Kariba and Cahora Bassa dams used to replace the historical time series of observed releases. This is necessary because the observed releases do not extend throughout the total duration of the modelling period (the dams did not exist in the earlier years).

### 5.1 Delta change ranges for rainfall

**Fig. 2:**
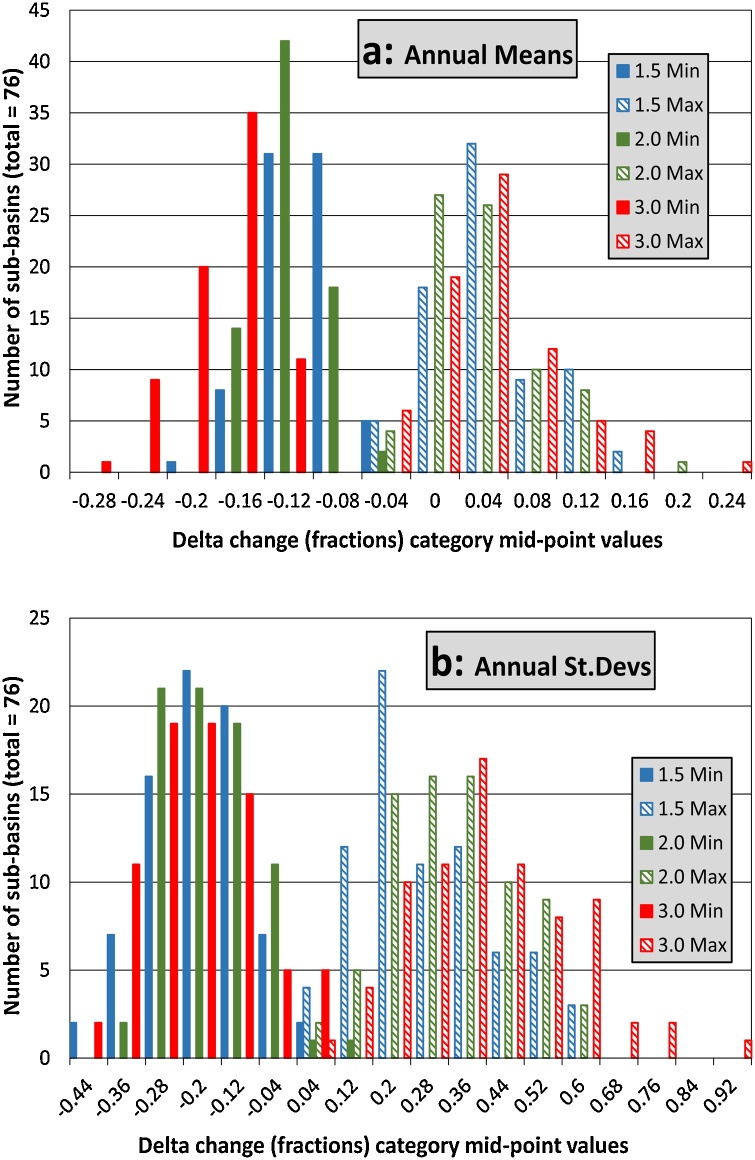
Frequency distributions of fractional delta change minimum (drier or less variable conditions) and maximum (wetter or more variable conditions) values for rainfall across the sub-basins; a) Annual means, and b) Annual standard deviations.

**Fig. 3:**
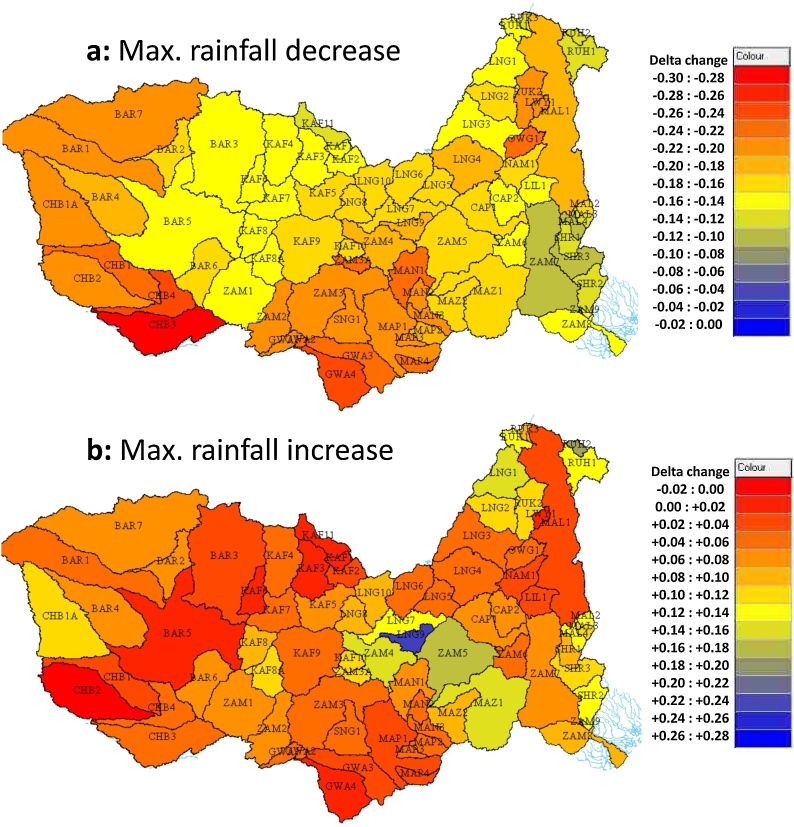
Spatial distribution of a) minimum rainfall changes, and b) maximum rainfall changes (the values are based on the maximum and minimums across all climate models and across the three global warming scenarios).

Fig. 2 shows the frequency distributions across all of the sub-basins for the annual means and standard deviations (minimum and maximum values representing the ranges of delta change across the six climate models), while Fig. 3 shows the spatial distribution of the maximum and minimum delta change in mean annual rainfall. The overall (across all three warming scenarios) spatial median for the annual mean minimum fractional delta (maximum decrease in rainfall) is approximately -0.13, while the equivalent value for the maximum increase is 0.03. The climate models therefore suggest a greater drying trend than a wetter trend. In terms of the annual standard deviation the median decrease is -0.125, while the median increase is 0.325, suggesting a trend towards more variable annual rainfall.

**Fig. 4:**
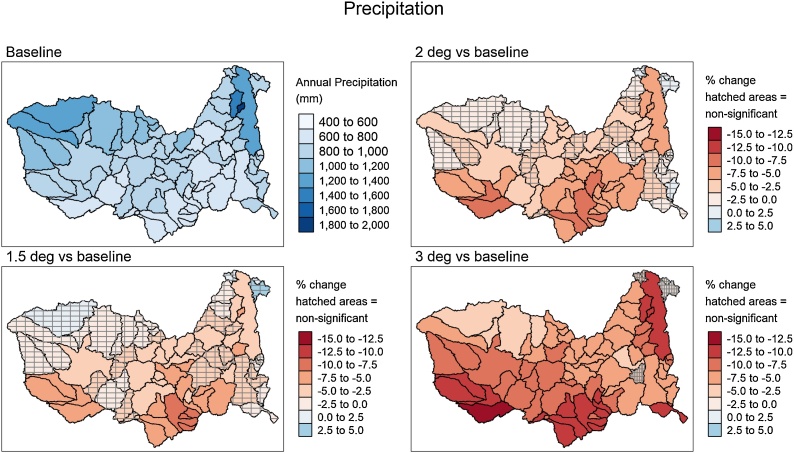
Spatial distribution of the annual precipitation and changes for the three global warming scenarios. The data plotted are based on the mean values for the six climate models used in this study (Table 2).

Fig. 4 illustrates the % changes, using the mean delta change across the climate models (with respect to their own baseline conditions), for the three global warming scenarios and supports the data presented in Fig. 2 that suggests that the differences between the 1.5° to 2.0° scenarios are substantially less than the changes for the 3° scenario. The spatial patterns suggest that there are more sub-basins in the southern and western parts of the catchment with the highest rainfall reductions, but Lake Malawi/Nyasa also shows quite high reductions if the climate model mean values are used.

### 5.2 Delta change ranges for potential evapotranspiration and temperature

**Fig. 5:**
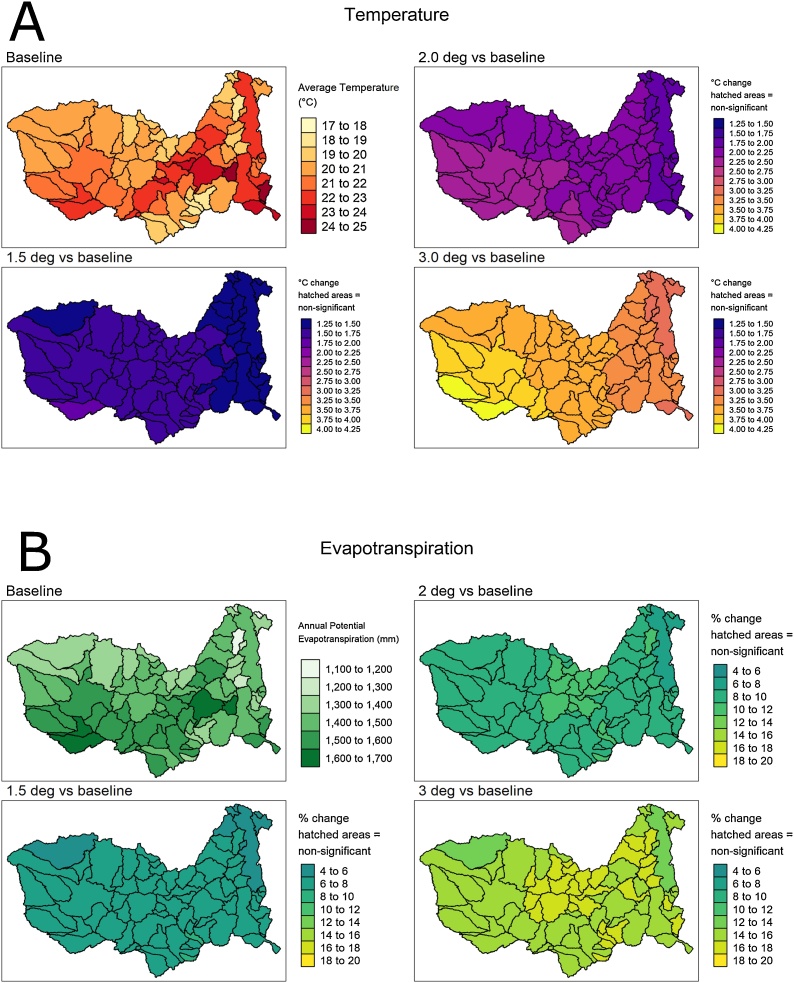
Spatial distribution of the annual temperature (A) and potential evapotranspiration (B) and changes for the three global warming scenarios. The data plotted are based on the mean values for the six climate models used in this study (Table 2).

Table 6 lists some statistics for the range of changes across the different climate models (lowest to highest) and across all of the sub-basins (mean, standard deviation, minimum and maximum). Fig. 5 (a and b) provides some indications of the spatial variability of the temperature and potential evapotranspiration changes expected for the three global warming scenarios (based on the climate model mean changes from their own baseline period).

**Table 6:** Changes in evapotranspiration demand for the three temperature change scenarios (expressed as a % of historical or baseline values) across all the sub-basins (‘Lowest’ and ‘Highest’ refers to the range across the different climate models).

Statistic	S2.2B		S3.2B		S4.2B	
	Lowest	Highest	Lowest	Highest	Lowest	Highest
Mean	102.6	111.1	102.3	114.9	108.8	120.8
St. Dev.	0.84	1.07	0.69	0.91	0.99	1.31
Minimum	100.3	108.3	102.3	111.9	108.8	116.7
Maximum	103.8	113.1	105.1	116.8	112.7	123.3

Fig. 5a highlights how the temperature changes in the basin are relatively higher than the global warming scenarios considered. Both Table 6 and Fig. 5b suggest that there is a relatively low degree of variation in terms of evapotranspiration demand in the changes across the sub-basins, although this increases for the 3.0° global warming scenario (S4.2B). Similarly, there are bigger overall differences between the 2.0° and 3.0° scenarios than between the 1.5° and 2.0° scenarios, a result that could be clearly anticipated and is also reflected in the rainfall data. There is no strong spatial pattern in the changes, partly a consequence of the relatively low variability across the sub-basins. However, it is interesting to note that the lowest changes for the 3° change occurs over the Lake Malawi/Nyasa sub-basin (MAL1), as well the upper Zambezi above the Barotse wetland (BAR2, BAR3 and BAR7). Most of the biggest changes occur in the lower sub-basins of the Luangwa and Kafue basins, as well as some of the Lake Malawi/Nyasa tributary sub-basins. This spatial pattern is repeated for all the global warming scenarios if we use the highest change estimates across the climate models, but different patterns emerge when the lowest estimates across the climate models are used. While these results reflect the results of the regional downscaling of the different climate models, the overall impacts on the modelling results is expected to be relatively small due to the low variability across the different sub-basins (Table 6).

### 5.3 Simulation results for scenarios 1A and 1B

The total present day consumptive use referred to in Table 5 amounts to a total of 1 532 * 10^6^ m^3^, which is 78 % less than the value given in ZAMCOM (2019), but nevertheless the same order of magnitude relative to the total water resources availability. The impact of including these revised estimates as part of the scenario using historical climate data (S1A) on the flow duration curves of the key stations (KAF7, MAL1, ZAM2 and ZAM6) is almost negligible. Even in those tributary basins where the water use appears to be quite high (Luangwa and the Zimbabwean sub-basins) the largest impacts are generally no greater than a 5% reduction in mean monthly flow volume. Scenario 1B is based on uncertainty ranges of expected future water use (and distributed dam volumes and catchment areas). The lower values of the range are based on the values provided in Table 5, column 3, while the upper ranges are variable depending on the degree of uncertainty in the future estimates. It is acknowledged that most of the future estimates are uncertain, but some (e.g. LNG10) are based on more information than others. The total for the values given in Table 5 is 3 330 * 10^6^ m^3^, while the total for the upper range of uncertainty is 3 950 * 10^6^ m^3^, representing increases of between 217 % and 258 % over the present day estimates. Even with the upper uncertainty estimates of water use, there are very few differences between the flow duration curves at the four key sites. The four Zimbabwean basins (GWA1, MAP1, MAN1 and MAZ1) show the largest impacts ranging from about a 90 % reduction at MAZ1 to almost a 70 % reduction at MAN1 for the upper uncertainty bound. The overall conclusion is that the amount of estimated consumptive water use has relatively little impact on the total resources of the Zambezi River.

### 5.4 Simulation results for scenarios 2.1 and 2.2

**Fig. 6:**
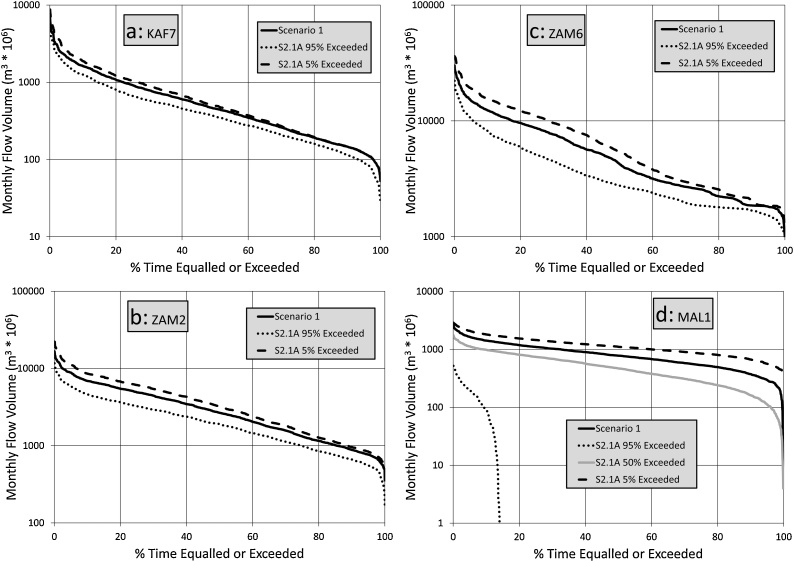
Flow duration curve comparisons between Scenario 1 and the 95 % and 5% exceeded ensemble bounds for Scenario 2.1A.

Fig. 6 shows the results for only the 1.5° temperature increase rainfall changes (S2.1A) for the selected sub-basins and it is quite clear that the 95 % exceeded impacts (the ‘worse’ case rainfall change ensemble) are quite different throughout the basin. The increased impacts noted in ZAM2 and ZAM6, compared to KAF7, are likely to be related to the larger rainfall reductions in the many of the headwater sub-areas of the upper Zambezi, relative to the Kafue headwaters (Fig. 3, Fig. 4), as well as the impacts of the combined effects of climate change on the Barotse floodplain wetland upstream (in BAR5). The result for MAL1 is very uncertain, partly because the original model calibration of the dynamics of Lake Malawi/Nyasa was very uncertain (Hughes et al., 2020). Some of the model calibrations for the contributing sub-areas were also quite uncertain. At least some of the higher degree of change (relative to the other sub-basins) might be attributed to the direct impacts of rainfall on the large surface area of the lake and the dominant role that this plays in the overall water balance. However, further analysis suggests that the total upstream inflow is also quite highly reduced in the driest ensembles. Attempts to attribute differences in flow reductions to the way in which the total runoff has been simulated did not reveal any clear patterns that might point to excessive reductions being linked to modelling artifacts. However, as might be expected, some of the sub-basins with high proportions of total runoff being simulated as surface runoff are linked to relatively higher stream flow reductions. This is particularly true if the majority of the surface runoff is simulated as saturation excess (rather than infiltration excess) runoff. In the former, the effects of reduced rainfall are not only direct (less rainfall to runoff), but also indirect and related to reduced moisture storage and therefore generally lower proportions of saturation.

It is quite difficult to assess whether the rather drastic reduction in outflow from Lake Malawi/Nyasa, and extended periods of no outflow, under the most extreme rainfall reduction ensemble, is a credible result or not. The results are in broad agreement with those of Kling et al. (2015), but some of other literature on climate impacts on the lake outflows do not always agree with each other, even in terms of the historical patterns of lake levels. Both Lyons et al. (2010) and Kumambala and Ervine (2010) used models to simulate the historical patterns of lake levels (and both refer to earlier papers covering a similar subject). However, they disagree quite substantially about the key water balance components of the basin and lake system, suggesting that it is difficult to use their results to check our own simulations. The CRU rainfall data used in this study suggest that the historical rainfall for the lake sub-basin is some 1 202 mm y^−1^, with the evaporation being 1 527 mm y^−1^ (a net loss of 325 mm y^−1^). Kumambala and Ervine (2010) suggest that the mean annual rainfall is actually at least 1 350 mm y^−1^, and evaporation about 1 610 mm y^−1^ (a net loss of 260 mm y^−1^). Further, Calder et al. (1995) noted that changes in rainfall can explain historical patterns of change in lake levels better than any other effects, but their analysis did not include any of the quite large changes in evaporation associated with a warming climate. We could not achieve a satisfactory calibration (relative to observed outflow patterns) in our model if we scaled our input data to give a net evaporative loss of 260 mm y^−1^ over the lake. The 50 % exceeded (median) ensemble has been included in Fig. 6 for the Lake Malawi/Nyasa outflows (MAL1) to illustrate that there is a substantial degree of non-linearity in the stream flow reductions compared to the rainfall reductions (which are based on uniform sampling).

**Fig. 7:**
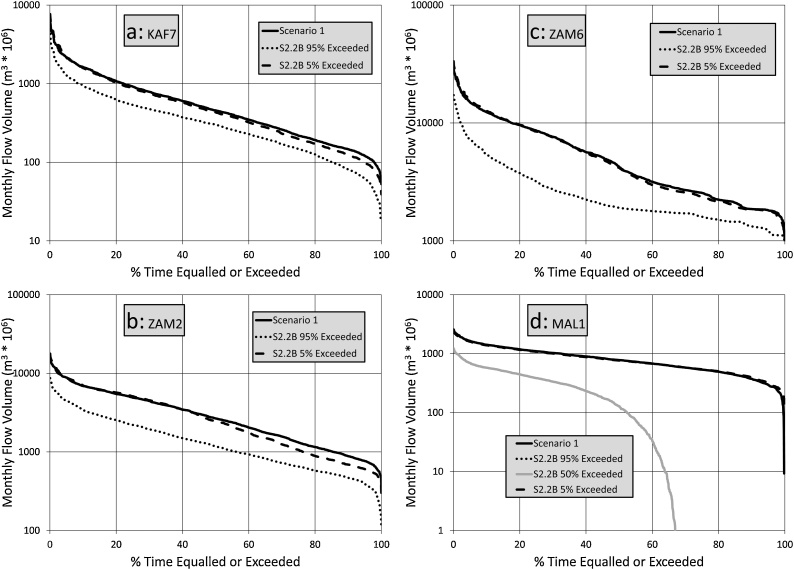
Flow duration curve comparisons between Scenario 1 and the 95 % and 5% exceeded ensemble bounds for Scenarios 2.2B.

Fig. 7 suggests that the inclusion of evaporation effects (S2.2B) for a 1.5° temperature change does not make a huge difference to the results at KAF7 and ZAM2, but does quite substantially reduce the moderate to high flows at ZAM6. As might be expected from the comments already made about Lake Malawi/Nyasa, the impacts of the drier ensembles (50 % exceeded or greater) on MAL1 are extremely large, with zero outflows for 100 % of the time in the 95 % exceeded ensemble. These results clearly reflect the increased impacts of evaporative losses downstream of large open water bodies (reservoirs and wetlands).

### 5.5 Simulation results for scenario 3.2B

**Fig. 8:**
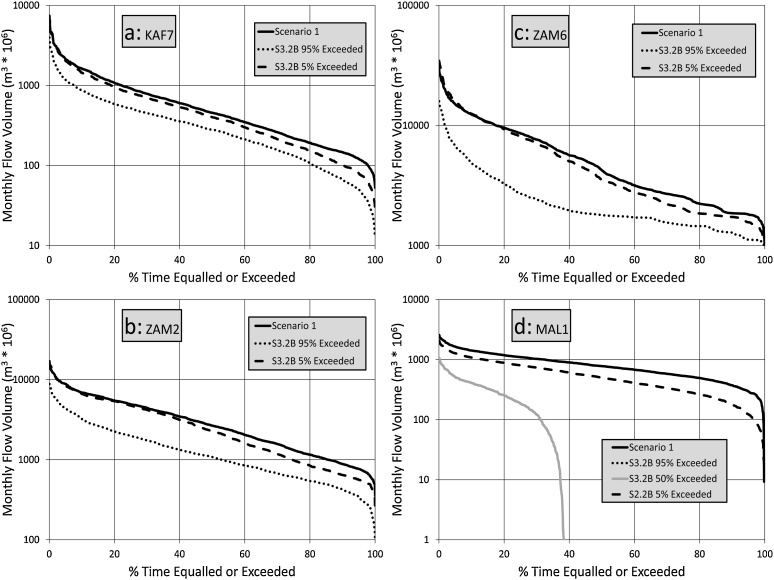
Flow duration curve comparisons between Scenario 1 and the 95 % and 5% exceeded ensemble bounds for Scenarios 3.2B.

Fig. 8 illustrates that the differences between S2.2B and S2.3B are not very large for most of the sub-basins, although the frequency of zero outflows for Lake Malawi/Nyasa increases quite substantially. This is associated with the relatively small differences between the delta change values for these two scenarios, while the differences in evaporation demand are also not very large when taking into account the uncertainty range, as well as the variations across the sub-basins.

### 5.6 Simulation results for scenario 4.2B

**Fig. 9:**
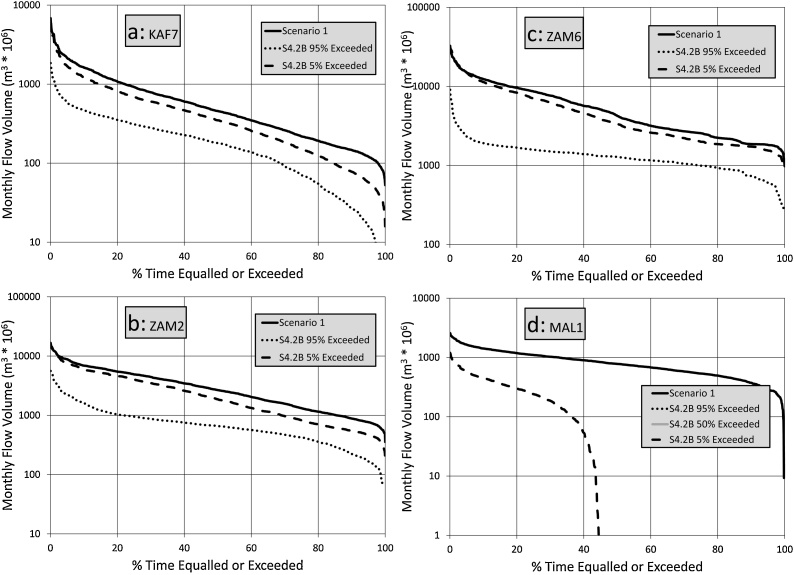
Flow duration curve comparisons between Scenario 1 and the 95 % and 5% exceeded ensemble bounds for Scenarios 4.2B (note the change in vertical axis range for ZAM1 and ZAM2 compared with Figs. 4 to 6).

Fig. 9 shows that the full complement of climate and water use change signals for a 3.0° temperature increase suggest a much greater degree of change for all of the sub-basins, but especially for the downstream parts of the main Zambezi River (ZAM6) and for Lake Malawi/Nyasa (MAL1). The results for ZAM6 are likely to be quite strongly affected by the operating rules of Kariba and Cahora Bassa Dams. No attempts have been made to optimize these for different climate scenarios and they have remained fixed for all of the model runs from scenario 1 onwards (see next section). For Lake Malawi/Nyasa, even the 5% exceeded scenario suggests that their will only be flow out of the lake into the Shire River for some 40 % of the time. To further examine the validity of this results, the mean annual water balance of Lake Malawi/Nyasa for the three main climate scenarios (S2.2B, S32.B and S4.2B) can be compared to the S1 scenario. The net evaporation from the lake (rainfall – evaporation) for scenario 1B is some 325 mm, which is balanced in the same scenario by about 760 mm of inflow. The net evaporation loss increases to a maximum (i.e. the lowest rainfall and highest evaporation estimates for each scenario) of 597, 613 and 746 mm for scenarios 2.2B, 3.2B and 4.2B, respectively. Given that the inflows progressively reduce through the three scenarios to less than 460 mm (for the overall driest situation), the graphs in Figs. 7d, 8 d and 9 d are not at all surprising.

### 5.7 Simulation results for Kariba dam

**Fig. 10:**
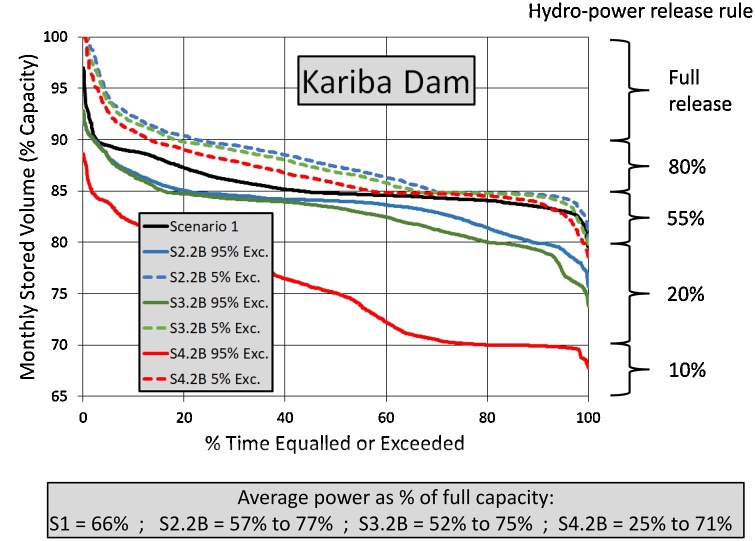
Exceedance frequency distribution of the of Kariba Dam storage volumes comparing the three main global warming and future water use scenarios with baseline.

Fig. 10 shows the exceedance frequency distribution of Kariba Dam storage volumes (expressed as a % of full storage capacity) for the three main temperature change and future water use scenarios (S2.2B, S3.2B and S4.2B) compared to the baseline scenario (S1). The 65 % level represents the approximate level below which hydro-power generation is no longer possible, while the ‘Hydro-power release rule’ column to the right of the graph shows the rules that have been implemented in all of the model runs. These rules were roughly based on the historical record of hydro-power releases that were used during the model calibration run, however, no attempt has been made to optimize them for any particular inflow regime. They are therefore only used to illustrate the likely differences in releases (and by implication, power generation) between scenarios. The text box at the bottom of Fig. 10 summarises the average release for each scenario relative to the full release capacity. The lower and upper values for the three change scenarios represent the 95 % and 5% exceedance values. The 5% exceedance values show either slightly increased, or very similar, releases relative to S1, while the worst case situation for the three scenarios show decreases of 12 %, 17 % and 40 % for the 1.5°, 2.0° and 3.0° temperature changes, respectively. No attempt has been made in this study to convert the release changes into actual power production, as the focus here is mainly on the water resources changes. Part of the complexity of the relationships between this diagram and the Kariba inflow changes at ZAM2 (Figs. 7d, 8 d and 9 d) are related to the fact that the modelled operating rules for Kariba Dam also include flood releases (dominantly at storage volumes of 90 % and higher), and their patterns of variability will also change across the various scenarios.

### 5.8 Changes in some headwater sub-basins

Table 7 compares the mean annual runoff volumes for scenario 1 with the other scenarios for selected headwater sub-basins with different aridity indices (mean annual potential evapotranspiration divided by mean annual rainfall), using the median of the ensemble output results. The results are inevitably quite mixed, partly because each sub-basin has a different range of rainfall and evapotranspiration delta changes, as well as water use changes. The original idea behind this comparison was to try and identify if different degrees of aridity and/or proportions of the three main runoff components in the calibrated model would influence the degree of change. For example, it might be expected that sub-basins dominated by saturated area surface runoff could experience greater impacts due to the combined effects of reduced rainfall (less runoff) and increased evapotranspiration (reduced moisture storage). However, there are too many other variables involved to reach any conclusions about these issues. Most of the differences can be explained by the range of rainfall delta changes across the three scenarios. For example, for RUH2 the upper values of the delta changes are positive and quite large for all three scenarios (0.18, 0.19 and 0.17), while for RUK2 they are weakly positive for S2.2B (0.11) and S3.2B (0.03), but negative for S4.2B (-0.02).

**Table 7:** Impact of rainfall and evapotranspiration demand changes for sub-areas with different aridity indices.

	Sub-basin (Aridity Index)				
	LWY1 (1.004)	RUH2 (1.038)	RUK2 (1.427)	MAZ2 (1.686)	GWA3 (2.123)
Runoff components for S1 (%)	SQ: 27	SQ: 15	SQ: 56	SQ: 95	SQ: 95
	IQ: 44	IQ: 61	IQ: 23	IQ: 0	IQ: 0
	GQ: 29	GQ: 24	GQ: 21	GQ: 5	GQ: 5
Scenario	Mean Annual Runoff (m^3^ * 10^6^) for Median Ensemble (% of S1 value)				
Scenario 1	1059.2	1151.7	1641.1	1434.6	469.0
Scenario 2.1A	884.9 (84 %)	1187.2 (103 %)	1530.4 (93 %)	1177.6 (82 %)	365.6 (78 %)
Scenario 2.2A	829.8 (78 %)	1153.5 (100 %)	1329.2 (81 %)	1059.3 (74 %)	341.0 (73 %)
Scenario 2.2B	829.7 (78 %)	1153.5 (100 %)	1329.2 (81 %)	998.3 (70 %)	325.2 (69 %)
Scenario 3.2B	836.7 (79 %)	1184.6 (103 %)	1148.3 (70 %)	1017.8 (71 %)	330.5 (70 %)
Scenario 4.2B	678.9 (64 %)	1113.0 (97 %)	745.7 (45 %)	941.3 (66 %)	317.3 (68 %)

*Notes*: Runoff components: SQ = surface runoff, IQ = interflow, GQ = groundwater contributions.

### 5.9 Discussion

Perhaps, one of the key questions for discussion is the extent to which the future changes are partly artefacts of the hydrological model setup and whether the climate change signals from the RCMs are being propagated appropriately through the model. In most senses it is reasonable to assume that this is not the case because the model has adequately simulated the runoff responses to quite a high degree of variability (both seasonally and inter-annually) in the historical record. However, given the high degree of uncertainty in our understanding of the dynamics of the large wetland systems (notably Lake Malawi/Nyasa), as well as the methods used to represent them in the model, we cannot entirely reject the possibility that some of the extreme changes that have been simulated are not, at least partly, modelling artefacts. Kumambala and Ervine (2010) predicted drops in lake levels using HADCM3 climate data, but do not predict zero outflows. However, their future climate inputs are quite different to those used in the present study and include higher rainfalls and quite modest increases in evaporation demand. The relatively simple water balance check that was included within the results section, suggest that the loss of downstream outflow from Lake Malawi/Nyasa is inevitable, given the climate change signals used in this paper and are certainly not a modelling artefact.

A related issue is associated with the fact that none of the hydrological parameters were changed from the historical calibrations to the climate scenario runs. While this may not be strictly the correct approach to adopt, we currently have little understanding, or scientific basis, for determining which parameters to change and by how much. We could have speculated about changes in such as infiltration, rooting depth or vegetation cover regimes and introduced uncertainty ranges into the relevant parameters as part of the future scenario ensemble runs, but these changes would not have been based on any real knowledge or full understanding. Such changes can be made to the model in the future if and when there is greater evidence for quantifying the changes.

It has already been noted that there are quite large discrepancies between our own uncertain estimates of future water use and those that are contained in previous reports. ZAMCOM (2019) refers to a more than threefold increase in consumptive water use, as well as total current use that is some 26 % higher than our own estimates. Kling et al. (2014), using figures based on a World Bank study, refer to current use that is 73 % higher than our values, as well as moderate and high development options that are some 2.4 and 7.6 times their current use estimates, respectively. However, on the one hand the sources of these other estimates are not fully explained, while on the other hand even a much greater increase in water use would have relatively small impacts compared to the effects of the lower bound estimates of the climate change signals. Despite this observation, it is acknowledged that our own analysis of water consumption patterns and change is based on weak information and should certainly be improved for future model runs. However, we have accounted for some of the constraints on development, such as competing users (irrigation and hydro-power in LNG8 and 10, for example), as well as competition for land resources between commercial irrigation and community rain-fed agriculture (both issues noted as important by Lawrence, 2020). There are also quite large tracts of land within some sub-basins of inter alia the Luangwa, Kafue and Chobe River sub-systems that are occupied by national parks that are considered here unlikely to experience substantial development impacts. Some of the smaller water uses (e.g. rural water supplies) have been largely ignored as likely to have very small impacts on the main points of interest within the basin. However, these could have significant impacts at a more local scale, particularly in the more arid parts of the basin, while small scale, but extensive use of groundwater could impact on the low flow regimes of some of the tributary sub-basins. Unfortunately, the necessary data are not currently available and therefore were not included.

The results show very clearly that the major source of uncertainty for the future lies in the projections of future climate conditions and these are heavily dependent upon all the many assumptions that are built into the GCMs RCMs, as well as the associated downscaling approaches. These are issues that hydrological modellers have little control over and we have to accept that our climate modelling colleagues generate the best information that they can (Hargreaves and Annan, 2014). The validity of the simulated stream flow changes is therefore very dependent upon the validity (or appropriateness) of the climate change signals used to force the model, given that the model responds correctly to the changed climate signals. The previous discussion paragraphs have partly addressed this issue, but it is also interesting to look quite broadly at the runoff drivers in this basin. There is a quite strong relationship between the aridity index (mean annual values of potential evapotranspiration divide by precipitation) and the runoff ratio for the headwater sub-basins (R^2^ = 0.65). If the aridity index values for the wetter and drier bounds of the three warming scenarios are re-calculated, the wetter bounds for all three scenarios show slight decreases or increases in aridity. However, the drier bounds (i.e. largest decreases in rainfall and largest increases in ET_0_) show increases in aridity of 124, 130 and 147 % averaged across all sub-basins for the three scenarios, respectively. Thus, the combined effects of reduced rainfall and reduced runoff ratio will certainly account for the results illustrated in Figs. 7d, 8 d and 9 d, particularly when the effects of increased evaporation losses from the open water bodies (reservoirs, Lake Malawi/Nyasa and the wetlands during the wet season) are also taken into consideration.

Kling et al. (2014) used two climate models (CNRM and MPI) and two temperature change scenarios (1.7° and 4.8°) and assessed the impacts upstream of Tete (ZAM6 in our model). Using the CNRM data produced higher flows for both futures, while with MRI the warmer temperature future produced results that were somewhat lower than the 5% exceeded line given in Fig. 9c. While Kling et al. (2014) do not provide very much detail of the climate change data used, their ‘climate sensitivity scenario’ is based on changes of ±10 % rainfall and temperature increases of 2° and 4°. Based on the changes in reservoir evaporation reported in the paper, the temperature changes translate into evaporation demand increases of between 4% and 8%. Fig. 2, Fig. 3, Fig. 4, Fig. 5 of this paper indicate that the possible decreases in rainfall and increases in ET_0_ used in this study are far greater for the 3° warming scenario. The difference in the projections of future water availability between their study and ours are therefore not at all surprising.

Comparisons with the results given by Beck and Bernauer (2011) are quite difficult as they lump together the impacts of development and climate change and have based their future simulations on very different assumptions from those used in this paper. The values that they give for development changes suggest a 16 times increase in consumptive water use (excluding evaporation from hydro-power dams). This increase is clearly far greater than any changes that we have accounted for and will clearly dominate the future values in at least some parts of the basin. It is perhaps not surprising that their worst case scenario predicts zero minimum flows at Victoria Falls (ZAM2) for all but 2 months of the wet season. It is similarly difficult to compare our results with those presented by Spalding-Fecher et al. (2017) because their results are confined to changes in power generation. However, for some of their drier scenarios they suggest that the average annual power availability at Kariba could drop to some 25 % of maximum generation capacity, which is similar to our worst case situation under scenario 4.2B (Fig. 10).

From a water management perspective, the bands of future uncertainty probably remain too wide in some parts of the basin for effective decision making and this further emphasizes the need to narrow the gap between the projections of the future climate forcing data. This contribution has not examined the sustainability of projected future water demands in any detail (apart from noting some possible changes in the power output of Kariba Dam in Fig. 10), largely because this was out of the scope of this study. However, the model setup can be used for this purpose, by simply looking at how frequently the projected demands are met under different change scenarios.

## 6 Conclusions

Hughes et al. (2020) and this study have attempted to demonstrate that the established Pitman model is fit for purpose in terms of simulating historical and future water resources availability. It is acknowledged that the model contains uncertainties, as will all models based on imperfect forcing data, with imperfect structures, and imperfect observational data with which to calibrate and validate the model. The papers have therefore also attempted to identify the key uncertainties and their likely impacts on the practical value of the model setup, and so that future studies can be focused on reducing these uncertainties. However, we believe that the main uncertainties do not invalidate the overall message of possible water resources change that is conveyed by the model results.

It is always difficult to make comparisons with previous studies of climate and development change, partly because the science of climate change assessment is highly dynamic, and partly because previous papers do not always contain the full details of the climate or development change signals used. We have provided quite a lot of details of the estimates of present day and future consumptive water use in this paper, but the results tend to be very low (notably for the future) compared to previous studies (Beck and Bernauer, 2011; Kling et al., 2011; ZAMCOM, 2019). While relatively moderate increases in the upper values of assumed water use are unlikely to have substantial effects on the main results, if the values used by Beck and Bernauer (2011) are really appropriate, then the results will be quite different. These estimates therefore remain a substantial source of uncertainty in our results.

One of the key conclusions is that the future simulation results are hugely dependent upon the source of the climate change data and the change signals that are given by them. The sample RCM data (6 models) used in this study were selected from recently available information to be representative of many more model outputs that are currently available. The spread of climate change signals across these different models remains quite large and that spread is translated into the future uncertainties illustrated in the main results diagrams of this paper (Fig. 6, Fig. 7, Fig. 8, Fig. 9, Fig. 10). The key points selected (KAF7, ZAM2, ZAM6 and MAL1), illustrate that the relative impacts can be quite different across the whole Zambezi River basin, the greatest impacts being in the Lake Malawi/Nyasa sub-system (in agreement with Kling et al., 2015), as well as other areas containing large open water bodies (natural and man-made), that are very sensitive to the combined effects of increased aridity. Additional uncertainty might be related to the climate change projections experiment selected. The dynamical downscaling methodology of the CMIP5 climate projections used in this analysis (CORDEX), might be different from a statistical downscaling exercise of the same GCMs’ outputs, and both from the newly released CMIP6 projections. The quantification of the uncertainty derived by this might the focus of future research.

What of the future development and use of the model? There remain many improvements that could be made to the model, as well as several additional more detailed analyses. It is hoped that Hughes et al. (2020) and this contribution, as well as the availability of the existing model setup, may inspire other interested individuals or groups within the region to pursue further investigations and add value to the model. The paper concludes with a few suggestions for further work:

• Further assessment of the regional groundwater recharge patterns and refinement of this part of the model (might also include the addition of groundwater abstraction as part of the water uses).
• Assessments of possible climate change consequences (land cover, etc.) and how these could affect the model parameter sets, rather than simply fixing the parameters between the historical and future scenario runs.
• Integrating various rainfall databases to provide improved historical forcing data and overcome some of the potential problems with using the CRU data.
• Further analysis of the available observed stream flow data, where there are apparent anomalies (Hughes et al., 2020), to extend the amount of model calibration data.
• Extending the application of the WEAP model to include the effects of development and climate change to provide further information that can be compared with the Pitman model results.
• Developing a better understanding of the dynamics of water exchange between the large wetlands and river channels, to improve the representation of wetlands in the model.
• Further detailed assessment of expected future water uses and their sustainability (for different purposes) under future climates across the sub-basins in the system, notably the semi-arid and quite highly developed Zimbabwe sub-basins.
• Assessments of the risks of various management decisions in the face of future uncertainty at different spatial scales and for different water resources sectors.
• Updating the climate change projections as new results become available from the IPCC AR6 climate models.

## Software and data availability

The Pitman model is available as part of the SPATSIM modelling framework from https://www.ru.ac.za/iwr/research/spatsim/. Further details about the Pitman model are included in the documentation that can also be downloaded (see the Pitman_Guide.pptx file in the SPATSIM_V3/doc folder). The model setups (including the forcing data, parameter sets, simulation results, etc.) can be obtained on request from one of the authors, subject to some restrictions on the distribution of the observed streamflow data.

## Declaration of Competing Interest

The authors declare that they have no known competing financial interests or personal relationships that could have appeared to influence the work reported in this paper.

All authors have seen and approved the final version of the manuscript being submitted. They warrant that the article is the authors' original work, hasn't received prior publication and isn't under consideration for publication elsewhere.

## Appendix A Algorithms used in the rainfall delta change approach

The annual means (AM) and standard deviations (SD)_are calculated for two rainfall time series for each climate model (i), one for the baseline period (PB_i_) and one for a future period (PF_i_). The delta changes for each climate model (ΔAM_i_ and ΔSD_i_) are calculated from Eqs. A1 and A2 and the maximum (AM_max, SD_max) and minimum (AM_min, SD_min) delta changes are found for all climate models.

(A1) ΔAM_i_ = (AM_PF_i_ – AM_PB_i_) / AM_PB_i_

and

(A2) ΔSD_i_ = (SD_PF_i_ – SD_PB_i_) / SD_PB_i_

The maximum and minimum delta changes in mean seasonal distribution proportions (MPj_max and MPj_min) are determined in a similar manner, based on the mean monthly (MMj, j = 1–12) rainfalls for each period and climate model Eqs. A3 to A5).

(A3) MPj_PB_i_ = MMj_PB_i_ / AM_PB_i_

(A4) MPj_PF_i_ = MMj_PF_i_ / AM_PF_i_

(A5) ΔMP_ij_ = (MPj_PF_i_ - MPj_PB_i_)/MPj_PB_i_

Samples of the delta change values are generated for each ensemble of the 250 future rainfall predictions (PP_k_, k = 1–250), using independent random numbers (Rand_AMk_, Rand_SDk_ and Rand_MPjk_, all between 0 and 1) and applied to the historical rainfall data (PH) that were used in the initial calibration of the hydrological model.

(A6) ΔAM_k_ = AM_min + Rand_AMk_ * (AM_max – AM_min)

(A7) ΔSD_k_ = SD_min + Rand_SDk_ * (SD_max – SD_min)

(A8) ΔMP_jk_ = MPj_min + Rand_MPjk_ * (MPj_max – MP_jmin_)

Initially, the annual time series (PP_y_, where y is the year of the time series) for the predicted ensemble (k) is created from the time series of historical rainfalls (PH_y_):

(9) AM_PP_k_ = AM_PH * (1 + ΔAM_k_)

(10) SD_PP_k_ = SD_PH * (1 + ΔSD_k_)

(11) PP_yk_ = AM_PP_k_ + SD_PP_k_ * (PH_y_ – AM_PH)/SD_PH)

The annual rainfalls are then distributed into monthly rainfalls (MM_jyk_) based on the historical monthly proportions of annual rainfall for each year (MP_jy_ = MM_jy__PH / PH_y_) and the appropriate delta changes (ΔMP_jk_)

(12) MM_jyk_ = PP_yk_ * MP_jy_ * (1 + ΔMP_jk_)

The final step is to adjust the sum of the MM_jyk_ values to equal the annual values (PP_yk_), given that the sum of the delta changes for the monthly proportions may not necessarily add up to 1 in any individual year.

## Footnotes

1 Lawrence, D., 2020. Personal communication from Mr D Lawrence of Imagen Consulting (http://www.imagenconsulting.net/services.html), experienced in water resources assessments in Zambia.
